# Effect of Sampling Interval on Dependence Between Hybrid Parameters of Machined Surface Textures

**DOI:** 10.3390/ma18235394

**Published:** 2025-11-29

**Authors:** Pawel Pawlus, Rafal Reizer, Wieslaw Żelasko

**Affiliations:** 1 Faculty of Mechanical Engineering and Aeronautics, Rzeszow University of Technology, Powstancow Warszawy 8 Street, 35-959 Rzeszow, Poland; 2 Institute of Materials Engineering, Faculty of Exact and Technical Sciences, University of Rzeszow, Pigonia 1 Street, 35-310 Rzeszow, Poland; rreizer@ur.edu.pl; 3 Faculty of Mechanics and Technology, Rzeszow University of Technology, Kwiatkowskiego 4 Street, 37-450 Stalowa Wola, Poland; w.zelasko@prz.edu.pl

**Keywords:** surface texture, roughness amplitude, hybrid parameters, sampling interval, machining, white light interferometer

## Abstract

Surface topography affects the functional properties of machined surfaces. To assess these effects, special parameters were developed. Among them, the hybrid parameters rms. slope Sdq and the developed interfacial areal ratio Sdr are of high significance. However, these parameters are interrelated. Sdr can be estimated from Sdq using the special formula. The connections between these parameters were studied in this work depending on the sampling interval. Sixty-four surface topographies, after various machining treatments, were measured by white light interferometer Talysurf CCI Lite and analysed. The same measurement condition was performed for all surfaces. The application of the formula that interrelated the hybrid parameters was tested for different sampling intervals. It was found that the Sdq parameter was strongly linearly correlated with the Sdr parameter, independent of the sampling interval. The dependence between hybrid parameters had a nonlinear character. The estimation of the Sdr parameter based on the values of the Sdq parameter yielded very accurate results for smooth surfaces, independent of the sampling interval. Relative errors in estimating Sdq based on Sdr decreased as the sampling interval increased. High errors corresponded to surfaces with high roughness amplitude. A proposal was presented for sampling interval selection. Because hybrid parameters are interrelated, one parameter should be selected for the description of the areal surface texture. The Sdq parameter is recommended because of its lower sensitivity to measurement errors than the Sdr parameter.

## 1. Introduction

Surface topography is related to the functional properties of machine elements. It is particularly related to contact mechanics, sealing, friction, and lubricant retention [1,2]. It consists of form, waviness, and roughness. After the removal of form, surface texture exists. It can be divided into roughness and waviness. Surface waviness occurs on a larger scale than surface roughness and should be minimised. The recommended surface texture depends on the application of the machine element. Previously, surface roughness was analysed based on 2D profile measurements. This method is still used in industry. However, the surface topography is three-dimensional in nature. The analysis of 3D areal surface textures led to more accurate results compared to the profile study [3,4]. Surface roughness is assessed based on parameters, which were developed for both profiles [5] and areal surface textures [6,7]. The amplitude parameters are the most popular [1,2]. They are related to friction and wear [8,9]. The smooth surface has a high tendency to seize [10,11]. The high amplitude is related to a tendency to fatigue [12,13]. Roughness height affects surface performance in contact [14,15]. Average amplitude parameters are stable on engineering surfaces [1,2]. However, hybrid parameters are also of high significance. The ISO 25178-2 standard [6] contains the following two hybrid parameters: rms. slope (surface gradient) Sdq and the developed interfacial areal ratio Sdr. Hybrid parameters contain information on the amplitude and spatial surface properties. The effect of surface amplitude on surface slope seems to be greater than that of the main wavelength.

The rms. slope Sdq is calculated using the following equation:

(1) $$S_{dq}=\sqrt{\frac{1}{A}\iint_{A}\left[{\left(\frac{\partial z(x,y)}{\partial x}\right)}^{2}+{\left(\frac{\partial z(x,y)}{\partial y}\right)}^{2}\right]dxdy}$$

where *A*—the definition area; *z*—surface height in position *x*, *y*; and *x*, *y*—lengths in perpendicular directions.

The developed interfacial areal ratio Sdr is computed using the following formula:

(2) $$S_{dr}=\frac{1}{A}\left[\iint_{A}\left(\sqrt{\left[1+{\left(\frac{\partial z(x,y)}{\partial x}\right)}^{2}+{\left(\frac{\partial z(x,y)}{\partial y}\right)}^{2}\right]}-1\right)dxdy\right]$$

The rms. slope can be applied to assess surface anisotropy [16]. Surface slope is related to surface contact, friction, wear, light reflection, hydrodynamics, and spalling [17]. Various definitions of the plasticity based on the slope of the surface structure elements have been specified [18,19,20]. The plasticity index describes the surface inclination towards plastic deformation. Childs [21] established that the low-slope surfaces corresponded to elastic contact. Torrance [22] and Berglund et al. [23] obtained the relations between slope and coefficient of friction. Typically, a higher slope corresponded to higher friction. Elvasli et al. [24] found a substantial influence of surface slope on wear in lubricated and dry reciprocating sliding. The surface slope can be used for the description of surface wear because it characterizes both spatial and amplitude properties [25,26]. During low wear, the surface slope is usually reduced. Low wear corresponds to wear within the original surface topography. The Sdq parameter is convenient for controlling the cosmetic appearance of surfaces and in sealing applications [27].

The developed interfacial areal ratio Sdr is related to the surface’s ability to create adhesive joints [28,29,30]. When the Sdr parameter is larger, this ability is also higher. However, it can also be proportional to the Sdq parameter, because Sdq and Sdr are strongly interrelated [31]. The analysis of the Sdr parameter is important for coating, wettability, and electrical conductivity studies. Croll [32] believes that the Sdr parameter is connected with corrosion protection and coating adhesion.

Surface texture measurement is typically performed using optical methods from which white light interferometry [33] is the most popular. Optical methods replaced stylus contact profilometers mainly due to the much higher measurement speed. Measurement using the stylus tip is subject to errors related to the contact of the tip with the measured surface, the dimension of the stylus tip (mechanical filtration) [34,35], or the so-called stylus flight [36,37]. However, optical methods are sensitive to the other errors related to the presence of non-measured points [38,39] or outliers [40,41]. The errors in measuring the surface texture are also related to the stitching [42,43] and presence of high-frequency noise [44,45]. Inaccuracies in the measurement of surface texture can be caused by improper filtration [46,47] and digitisation [48].

Hybrid parameters are highly sensitive to mechanical filtration by stylus tip, stylus flight, surface stitching, and the presence of high-frequency noise [49]. Changes in the sampling interval cause great changes in the hybrid parameters. However, selecting the appropriate sampling interval (SI) is a problem of high importance.

The minimum sampling interval depends on the measurement method. For example, when the stylus tip is used, the SI should be greater than the size of the tip [50]. For optical instruments, the minimum SI depends on the unit beam spot [51]. For SIs that are too small, data are highly intercorrelated (redundant). For excessively large sampling intervals, spatial information can be lost. Whitehouse and Archard [26] proposed a sampling interval equal to the autocorrelation length (the distance at which the autocorrelation function decays to a value of 0.1). However, the SI determined by this way can be very high. Other researchers proposed smaller sampling intervals. The optimum SI can be selected based on the power spectral density function [48,52,53,54,55]. The selection of the SI depends on the purpose of the investigation. Poon and Bhushan [56] and Thomas and Rosen [57] considered surface deformation. Pawlus and Zelasko [58] studied the effect of the SI on the contact mechanics of rough surfaces. Blunt and Ebdon [59] considered the grain size on the grinding wheel surface in their selection of the SI. Chetwynd and Pawlus [60] took into account the recognition of deep valleys on the honed cylinder surfaces.

Based on the book [1], Sdr can be approximated from Sdq using the following equation:

(3) $$S_{dr}=\frac{S_{dq}^{2}}{2}$$

The question arises whether this relation is valid. The effect of the sampling interval on the dependence between the Sdq and Sdr hybrid parameters will be studied for a large number of measured areal surface textures.

## 2. Materials and Methods

A total of 64 surface topographies were measured using a Talysurf CCI Lite (Taylor Hobson Ltd., Leicester, UK) white light interferometer with a vertical resolution of 0.01 nm and a vertical range of 2.2 mm. Talysurf CCI Lite uses an external white light source, specifically, a 150 W quartz lamp. Before measurements were taken, the samples were cleaned in acetone. Three or four repetitions or measurements per sample were taken and representative textures were analysed. The same measurement condition was applied to all surfaces. Measurements were made using an objective lens with a magnification of 5× and a numerical aperture of 0.13. The sampling interval was 3.2 µm, the measuring area, containing 1024 × 1024 data points, was 3.29 mm × 3.29 mm. For curved surfaces, form was removed by the second-degree polynomial, and flat surfaces were levelled without the application of digital filters. The non-measured points were filled in. Outliers were eliminated by surface truncation [41] (thresholds were used to remove erroneous points) corresponding to material ratios between 0.01 and 99.99%.

Surface topographies after one-process honing (8 samples), plateau honing (8 samples), milling (8 samples), lapping or polishing (8 samples), grinding (8, samples), vapour blasting (8 samples), and vapour blasting followed by lapping (8 samples) and laser textured surfaces with isolated oil pockets (8 samples) were measured and analysed. An attempt was made to test various types of surfaces; therefore, two-process (having traces of two processes as follows: plateau honed, laser textured, and vapour blasted and lapped) and one-process (other surfaces), anisotropic (honed, plateau honed, milled and ground), isotropic and mixed (other surfaces), deterministic (milled surfaces), random-deterministic (laser-textured surfaces), and random (other surfaces) samples were measured and analysed. The values of the rms. height Sq parameter were between 0.1 and 9.1 µm. Honed and plateau honed (two-process) cylinder samples were made of grey cast iron of 218 HB hardness and a diameter of 130 mm. They are typically used in diesel engines for heavy commercial vehicles. They were made of pearlitic grey cast iron of grade 35 (ASTM A48). The rest of the samples were made from 42CrMo4 steel of 40 HRC hardness, obtained after heat treatment. Honed and plateau honed surfaces of the cylinder liners were measured; the other samples were flat.

The one-process cylinder liners were rough-honed using diamond stones D151 (D151/112/×44/35) or finish-honed with diamond D76 (D76/112/×44/25) or ceramic stones 8 × 10 × 150 GS150-60GV4S. The honing angle was in the range of 15.5–125°. Plateau honed cylinder liners were finished and then plateau-honed with diamond stones (D76 and D15/118/×44/75) or ceramic stones (8 × 10 × 150 GS150-60GV4S and 8 × 10 × 100 YC500-R5-V). The honing angle after plateau honing was 55°. Plateau honing was performed with different plateau honing times. HON15 fluid was the coolant. The milling process was carried out with a vertical milling machine with a 320 × 1300 mm table and a self-propelled beam with a swivel head in two planes. During rough milling, rotational speed was 500 rpm with a feed of 80 mm/min, and during finish milling, rotational speed was 800 rpm with a feed of 40 mm/min. The lapping was performed using a grinder with a rotational speed of 100 rpm perpendicular to the directionality of the machined surface and hand wet polishing using paper of 2000 µm granularity. The polishing process was carried out using a polisher with adjustable rotational speed, replaceable discs, and variable pressure between the disc and the polishing wheel. The surfaces were polished with a diamond suspension of 9 µm granularity (rough machining and 1 and 3 µm granularities (finish machining)). The grinding was performed using a flat surface grinder. The rotational spindle speed was 1500 rpm, and the transverse feed was 2000 mm/min for rough grinding and 1500 mm/min for finish grinding. Vapour blasting was performed in a special cabin. The distance between the nozzle and the machined discs and the angle of incidence of the stream were variable parameters. Laser texturing was carried out using a laser engraver. The engraver was equipped with an ytterbium fibre laser. Due to the laser characteristics, the beam parameters minimised material buildups around the oil pockets. The laser power was 20 W, the focal diameter and length were 64 µm and 254 mm, respectively, the pulse duration was 1.5 ns, the feed speed was 200 mm/s, and the pulse repetition rate was 820 kHz.

An attempt was made to obtain diversified surface textures following special machining processes.

The effect of the sampling interval on the dependence between hybrid parameters was studied in detail for surfaces A–H (Table 1).

The following parameters from the ISO 25178-2 standard were analysed: rms. height Sq, rms. slope Sdq, the developed interfacial areal ratio Sdr, skewness Ssk, correlation length Sal, and texture aspect ratio Str. Skewness Ssk depends on the shape of the ordinate distribution; for two-process surfaces, it is negative. The correlation length Sal is the smallest distance at which the autocorrelation function decays to the value of 0.2. The texture aspect ratio Str is the ratio of the smallest and the highest correlation lengths. For the isotropic surface, it is close to 1 and for the anisotropic surface near 0. The parameters Sq, Ssk, Sal, Str, Sdq, and Sdr describe the texture of the areal surface. The surface texture parameters were calculated using TalyMap 6 (Digital Surf, Besancon, France) software.

These parameters were calculated for sampling intervals of 3.2, 10, 20, 50, and 100 µm. The sampling intervals of 50 and 100 µm seem to be too large for practical application and considering the used evaluation area. They were analysed to show the tendency for changes in the hybrid parameters. Sampling interval increases were made using TalyMap software. The changes in the parameters of the areal surface texture were compared for various SIs. For these sampling intervals, the application of Formula (3) was tested. Additionally, for different sampling intervals, the values of the linear correlation coefficient between the Sdq and Sdr parameters were calculated.

**Figure 1:**
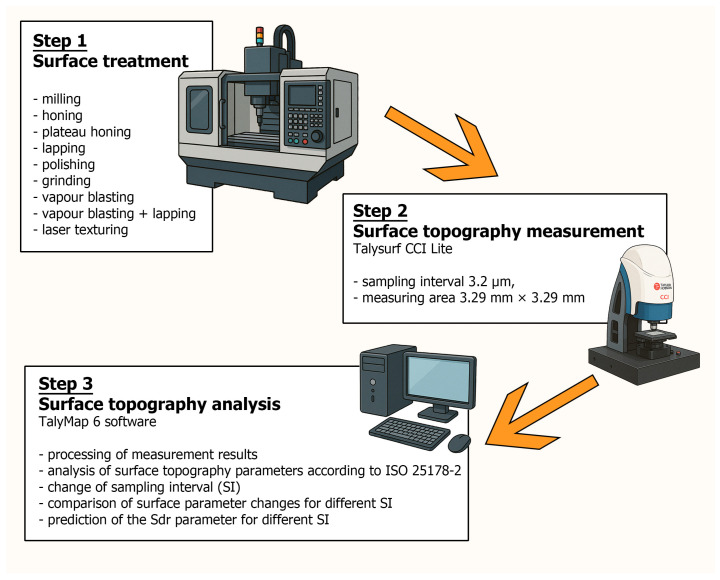
Scheme of our research.

Figure 1 presents the scheme of our research.

## 3. Results and Discussion

The surface textures after grinding were characterised by rms. height (Sq parameter) values between 0.22 and 1.12 µm (average value 0.54 µm). The correlation length Sal values were between 0.008 and 0.32 mm (average value 0.021 mm); the highest value corresponded to the roughest surface. The Str parameter was very small, between 0.019 and 0.052 (average value 0.033); therefore, the ground surfaces were anisotropic. The Ssk skewness was between −0.8 and −0.2 (average value −0.45). The Sdq parameter was between 0.08 and 0.18 (average value 0.11), and Sdr was between 0.29 and 1.6% (average value 0.68%)—the highest value corresponded to the highest roughness height defined by the Sq parameter. The shape of the autocorrelation function proved that the surfaces had a random character. Generally, ground surfaces were one-process, anisotropic (one-directional) surfaces with a slightly asymmetric ordinate distribution (negative values of the Ssk parameter) and moderate height.

**Figure 2:**
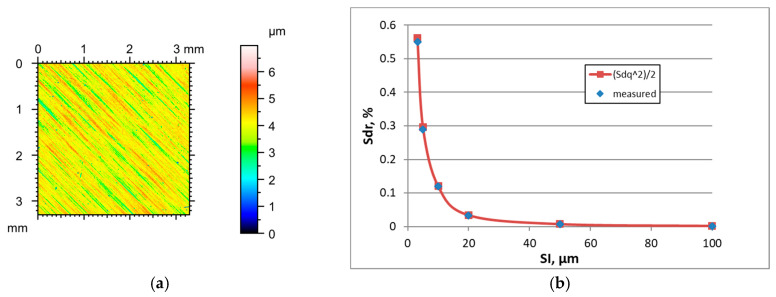
Height map (**a**) and changes in the Sdr parameter with an increase in the sampling interval (**b**) of milled surface A.

The increase in SI caused a decrease in the Sq parameter and an increase in the Sal parameter; the changes in Sq were smaller. The highest changes occurred for hybrid parameters, which decreased as the sampling interval increased. The parameters Ssk and Str were quite stable; they changed for sampling intervals of 50 or 100 µm; the Str parameter increased and the tendency for changes in the skewness Ssk was not clear. Figure 2 presents the height map for the smallest SI of the ground surface A characterised by an rms. height Sq of 0.48 µm. Table 2 shows the changes in the hybrid parameters of this surface caused by an increase in the SI. The application of Formula (3) caused an overestimation of the Sdr parameter; relative errors were the highest for the highest sampling interval. Similar results were obtained for other ground surfaces.

Surface textures after milling had deterministic natures. They were characterised by a high roughness amplitude, determined by the Sq parameter as follows: between 0.33 and 9.1 µm (average value 3.65 µm). The correlation length Sal was between 0.01 and 0.38 mm, and high values corresponded to rough surfaces (average value 0.13 mm). The texture aspect ratio was between 0.04 and 0.53 (average value was 0.19); therefore, the surfaces had an anisotropic (after fine milling) or mixed (after rough milling) character. The Ssk skewness range was between −0.7 and 0.5 (average value −0.09). The rms. slope Sdq was between 0.03 and 0.39 (average value 0.18) but Sdr was between 0.04 and 5.2% (average value 2.1%). One-process surfaces after milling had a deterministic character; they were anisotropic or mixed.

**Figure 3:**
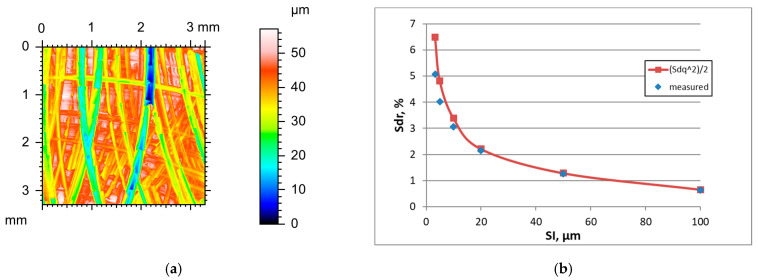
Height map (**a**) and changes in the Sdr parameter with an increase in the sampling interval (**b**) of milled surface B.

Unlike ground surfaces, the parameters of milled textures were rather stable in increasing the sampling interval when it was smaller than the correlation length Sal, except for the hybrid parameters which decreased. For excessively high SIs, the Sq parameter decreased and Str increased for initially anisotropic surfaces. Typically, the Sal parameter decreased with an increase in the sampling interval, different from ground textures. The tendency for the changes in the Ssk skewness was not clear. Figure 3 presents the height map for the smallest SI and the effect of the sampling interval on the Sdr parameter of milled surface B, characterised by a high amplitude (Sq = 9.1 µm). Table 3 shows changes in the hybrid parameters of this surface caused by an increase in the SI. For sampling intervals of 3.2, 5, and 10 µm, the application of Formula (3) yielded an overestimation of the Sdr parameter with relative errors of near 28, 20, and 10%, respectively. The errors decreased for higher SIs and were less than 2.1%. Similar results were found for other rough milled surfaces. For smoother surfaces after fine milling, low errors occurred in estimating the Sdr parameter for small sampling intervals.

The amplitude of surface textures after abrasive blasting characterised by the Sq parameter was high as follows: between 1.23 and 2.9 µm (average value 1.99 µm). Surfaces had an isotropic character; the Str parameter was between 0.74 and 0.88 (average value 0.81). The Sal parameter was between 0.009 and 0.25 mm (average value 0.017 mm). The Ssk skewness was between −0.7 and −0.4 (average value −0.54). The Sdq parameter was between 0.34 and 0.53 (average value 0.44) and Sdr between 5 and 11.7% (average value 8.26%). The highest values of the hybrid parameters resulted from the high amplitude and comparatively small correlation length. Generally, the random surfaces after abrasive blasting were one-process isotropic.

**Figure 4:**
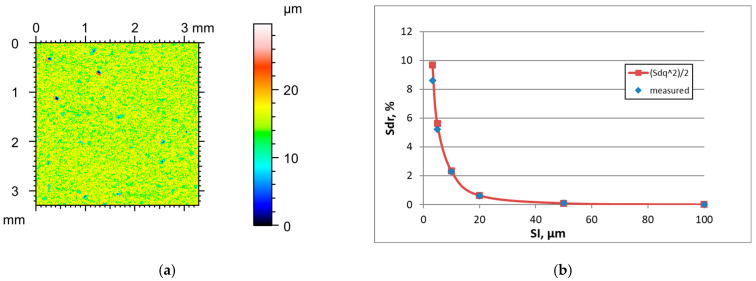
Height map (**a**) and changes in the Sdr parameter with an increase in the sampling interval (**b**) of vapour blasted surface C.

The increase in the sampling interval of surfaces after vapour blasting caused decreases in the Sdq, Sdr, and Sq parameters and an increase in the correlation length Sal. The texture aspect ratio Str decreased in most cases. The tendency for changes in skewness Ssk with changes in SI was not clear. Figure 4 presents the height map for the smallest SI, and the effect of the sampling interval on the Sdr parameter of the vapour blasted surface C characterised the initial value of the Sq parameter of 1.3 µm. Table 4 shows changes in the hybrid parameters of this surface caused by an increase in the SI. For the smallest SI of 3.2 µm, the application of Formula (3) led to a 12.7% overestimation and for a sampling interval of 5 µm to a 7.5% overestimation of the Sdr parameter. A further increase in the sampling interval led to an error of less than 3.3%. The results for other surfaces after vapour blasting were similar.

The amplitudes of one-process honed cylinder surfaces, characterised by the Sq parameter, were similar to those of the surfaces after vapour blasting, between 0.92 and 2.83 µm (average value 1.68 µm). Surfaces were anisotropic; the Str parameter was between 0.013 and 0.21 (average value 0.043). The correlation length Sal was between 0.018 and 0.035 mm (the mean value 0.023 mm). The highest values of the spatial parameters Sal and Str corresponded to the highest amplitude of roughness. The Ssk skewness ranged from −0.6 to 0.45 (average value −0.1). The Sdq parameter was between 0.15 and 0.29 (average value 0.22) and Sdr between 1.1 and 2.6% (average value 2.06%). The smaller values of the hybrid parameters of one-process honed surfaces compared to those after vapour blasting probably resulted from a slightly smaller amplitude and the anisotropic character of the honed textures. These surfaces were random, one-process anisotropic surfaces (with cross-hatched texture).

**Figure 5:**
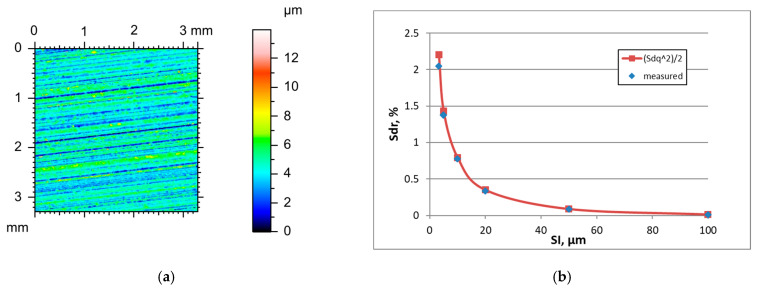
Height map (**a**) and changes in the Sdr parameter with an increase in the sampling interval (**b**) of one-process honed surface D.

Similarly to other analysed random surfaces, the increase in sampling interval caused a decrease in the Sq parameter and an increase in the correlation length Sal of one-process cylinder surfaces. For a large sampling interval, the Str parameter increased. The increase in the sampling interval caused a decrease in both hybrid parameters Sdq and Sdr, but the tendency for changes in the skewness Ssk was not clear. Figure 5 presents the height map for the smallest sampling interval and the effect of the sampling interval on the Sdr parameter of one-process honed surface D, characterised by the initial parameter of a Sq parameter of 1.38 µm. Table 5 shows the changes in the hybrid parameters of this surface caused by an increase in the SI. When the SI was 3.2 µm, the application of Formula (3) led to an overestimation of the Sdr parameter of about 8%. For higher sampling intervals, these overestimations were smaller than 4%.

Surfaces after polishing or lapping were characterised by the smallest roughness heights from the analysed textures, with Sq values between 0.01 and 0.06 µm (average value 0.034 µm). The correlation length Sal was comparatively low, between 0.004 and 0.01 mm (average value 0.0065 mm). The surfaces were mixed—the Str parameter was between 0.15 and 0.6 (average value 0.33). The skewness was between −0.32 and 0.14 (average value −0.16). Due to the smallest amplitude, the hybrid parameters were also the smallest—Sdq between 0.003 and 0.02 (average value 0.01) and Sdr between 0.0004 and 0.02% (average value 0.0078%) for all textures analysed. These surfaces were random one-process surfaces of a mixed isotropy character and with a very small amplitude.

**Figure 6:**
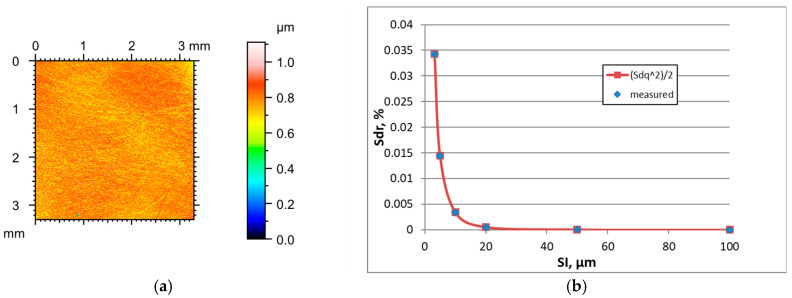
Height map (**a**) and changes in the Sdr parameter with an increase in the sampling interval (**b**) of lapped surface E.

An increase in the sampling interval led to decreases in Sdq, Sdr, and Sq and increase in the height Sq of the surfaces after lapping or polishing. The skewness Ssk and texture aspect ratio Str typically decreased when the SI increased. Figure 6 presents the height map for the smallest sampling interval and the effect of the sampling interval on the Sdr parameter of lapped surface E, characterised by the initial parameter of a Sq parameter of 0.06 µm. Table 6 shows the changes in the hybrid parameters of this surface caused by an increase in the SI. The application of Formula (3) led to an overestimation of the Sdr parameter. For a SI of 50 and 100 µm, the relative errors were greater than 5%.

Plateau honed surfaces were two-process cross-hatched texture surfaces obtained after rough honing followed by plateau honing. They were characterised by a high amplitude range, the Sq parameter was between 0.33 and 2.86 µm (average value 0.98 µm). The Sal parameter was between 0.013 and 0.047 mm (average value 0.023 mm). The surfaces were anisotropic—the Str parameter was between 0.01 and 0.077 (average value 0.034). The increase in the roughness amplitude led to an increase in the spatial parameters Sal and Str. Skewness Ssk was negative, between −3.2 and −1, which is a characteristic feature of two-process textures (average value −1.9). The parameter Sdq was between 0.09 and 0.26 (average value 0.16) and Sdr was between 0.43 and 2.96% (average value 1.4%). The plateau honed surfaces were two-process random anisotropic (cross-hatched) structures.

**Figure 7:**
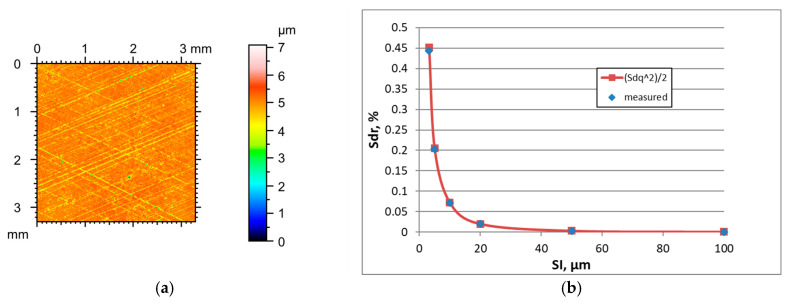
Height map (**a**) and changes in the Sdr parameter with ab increase in the sampling interval (**b**) of plateau honed surface F.

Similarly to other analysed surfaces, the increase in the sampling interval of plateau-honed cylinder surfaces caused the highest decreases in the hybrid parameters, a decrease in the roughness amplitude Sq, and an increase in the correlation length Sal. When the sampling interval increased, the texture aspect ratio Str increased. The tendency for changes in Ssk skewness during the SI change was not clear. Figure 7 presents the height map for the smallest sampling interval and the effect of the sampling interval on the Sdr parameter of plateau honed surface F characterized by the initial parameter of a Sq parameter of 0.33 µm. Table 7 shows the changes in the hybrid parameters of this surface caused by an increase in the SI. The application of Formula (3) caused an overestimation of the Sdr parameter. However, the relative errors were less than 3.2%.

The two-process surfaces after vapour blasting followed by lapping were characterised by negative values for the skewness Ssk, between −3.6 and −1.4 (average value −2.6). The Sq parameter was between 0.5 and 1.2 µm (average value 0.9 µm). The correlation length Sal was between 0.017 and 0.036 mm (average value 0.024 mm). The surfaces were isotropic as follows: the Str parameter was between 0.76 and 0.88 (average value 0.825). The rms. slope Sdq was between 0.12 and 0.2 (average value 0.16) and the Sdr parameter was between 0.67 and 1.73% (average value 1.3%). Hybrid parameters were similar to those obtained for plateau honed cylinder surfaces. The other analysed parameters of these two groups for these surfaces were also similar, except for Str. Two-process random surfaces after vapour blasting followed by lapping had isotropic textures.

**Figure 8:**
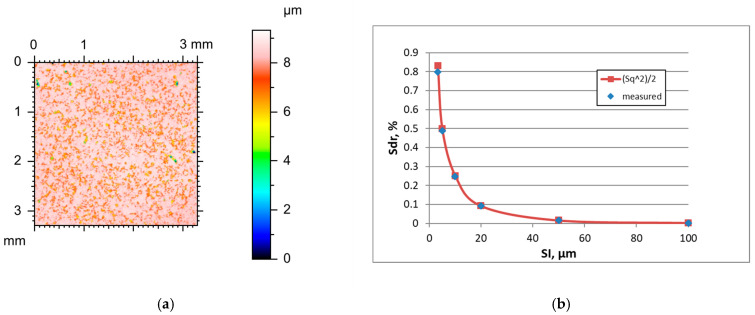
Height map (**a**) and changes in the Sdr parameter with an increase in the sampling interval (**b**) of surface G after vapour blasting followed by lapping.

Similarly to other analysed surfaces, the sampling interval increase caused decreases in hybrid parameters and rms. height Sq and an increase in the correlation length Sal of two-process surfaces after vapour blasting followed by lapping. The texture aspect ratio Str and typical skewness Ssk decreased when the SI increased. Figure 8 presents the height map for the smallest sampling interval and the effect of the sampling interval on the Sdr parameter of surface G, characterised by the initial parameter of a Sq parameter of 0.7 µm. Table 8 shows the changes in the hybrid parameters of this surface caused by an increase in the SI. The highest overestimation of the Sdr parameter using Formula (3) was found for the smallest sampling interval and was equal to 4.3%. An increase in the SI caused smaller errors in Sdr parameter estimation of up to 2.5%.

Random-deterministic textured surfaces with isolated oil pockets had a wide range of parameters. Oil pockets were created by a laser (deterministic part), and the surface between them was created by abrasive processes such as lapping, grinding, or honing (random part). Like other two-process textures, the Ssk parameter was negative, between −6.3 and −2.2 (average value 3.7). The roughness height determined by the Sq parameter was between 0.36 and 3.05 µm (average value 1.58 µm). The Sal parameter was between 0.05 and 0.26 mm (average value 0.125 mm). The Str parameter was between 0.46 and 0.88 (average value 0.72). The comparatively high values of spatial parameters are a consequence of the presence of oil pockets. The rms. slope Sdq was between 0.055 and 0.16 (average value 0.09) and the Sdr parameter was between 0.12 and 1.2% (average value 0.45%). The hybrid parameters were smaller compared to other two-process surfaces, which is the consequence of higher values of the correlation length Sal for laser-textured surfaces. These two-process surfaces of a random-deterministic character were typically isotropic.

**Figure 9:**
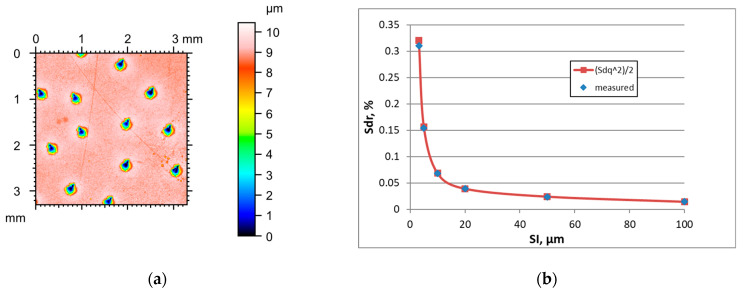
Height map (**a**) and changes in the Sdr parameter with an increase in the sampling interval (**b**) of surface H after laser texturing.

Similarly to other analysed surfaces, the increase in sampling intervals caused decreases in the hybrid parameters of laser-textured surfaces. Skewness Ssk changed marginally with the sampling interval change. The decrease in the Sq parameter with an increase in the SI was smaller than for random surfaces, similar to deterministic textures after milling. The texture aspect ratio Str decreased when the sampling interval decreased. The character of changes in the correlation length Sal with SI changes depended on the kind of surface. When the initial value of the Sal parameter was low (up to 0.05 mm), the increase in the sampling interval caused an increase in Sal. But for an initial Sal value close to 0.2 mm, an increase in the SI led to a decrease in Sal. Figure 9 presents the height map for the smallest sampling interval and the effect of the sampling interval on the Sdr parameter of the laser-textured surface H, characterised by the initial parameter of a Sq parameter of 1.23 µm. Table 9 shows the changes in the hybrid parameters of this surface caused by an increase in the SI. The application of Formula (3) led to overestimation of the Sdr parameter. The highest error, 3.2%, was obtained for the smallest SI, and for higher sampling intervals, the relative errors were smaller than 2%.

Regardless of the surface character, the increase in sampling interval caused decreases in both the hybrid parameters Sdq and Sdr. Generally, Formula (3) accurately predicted the character of the dependence between these parameters for small slope values. The surface height determined by the Sq parameter decreased when the sampling interval increased. The character of the changes in other parameters with an increasing sampling interval depended on the character of the surface. The parameters of deterministic surfaces after milling and random deterministic surfaces after laser texturing were more stable than those of random surfaces. For deterministic textures, the Sal parameter decreased when the SI increased, especially for comparatively high initial values of the correlation length. However, for random surfaces, the Sal parameter increased with an increase in the SI. The changes in the texture aspect ratio Str with the changes in the SI depended on the character of the surface. For anisotropic surfaces, Str increased, but for isotropic textures, Str decreased when the SI increased. In most cases, it was difficult to obtain a clear trend for the variations in the skewness Ssk with changes in sampling intervals.

**Figure 10:**
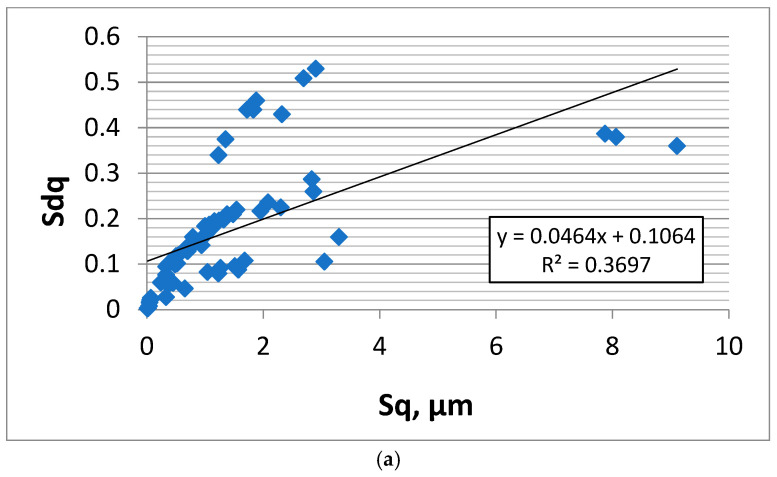

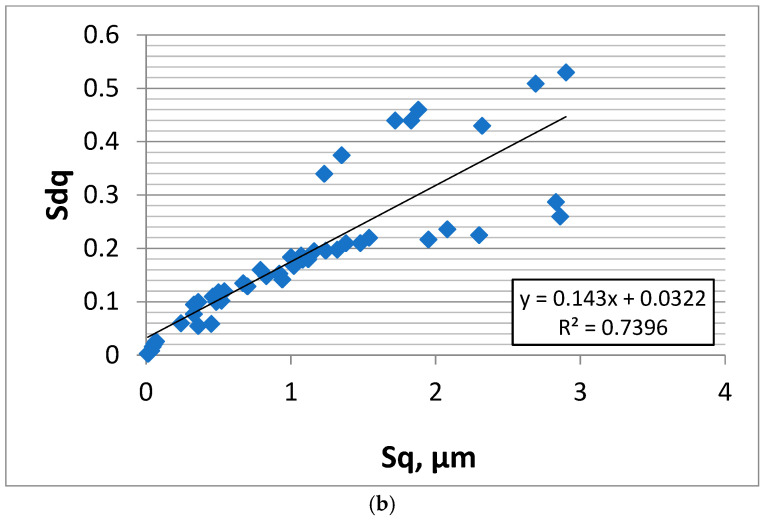
Dependence of Sq on Sdq for all surfaces (**a**) and for random surfaces (**b**).

The hybrid parameters depend on the amplitude and spatial properties of the surfaces. They are higher when the main wavelengths are smaller. The hybrid parameters of the laser-textured surfaces were smaller than those of other two-process surfaces because the correlation lengths of the textured surfaces were higher. The two-process surfaces after plateau honing and vapour blasting followed by lapping had similar rms. slope Sdq values because of similar Sal and Sq parameters, although the first group of surfaces was isotropic and the last anisotropic cross-hatched. However, it is difficult to obtain the effect of spatial parameters on the hybrid parameters for surfaces of various texture aspect ratio Str values. The two spatial parameters Sal and Str do not contain detailed information about surface character, such as whether the anisotropic surfaces are one-directional or cross-hatched. It seems that the effects of roughness amplitude on hybrid parameters are larger than the impact of spatial properties because of the high range of surface amplitudes of the analysed surfaces, characterised by the Sq parameter. Figure 10a shows the dependence of the Sq parameter on the initial rms. slope Sdq obtained for the smallest sampling interval for all analysed surfaces (64 data points). However, the correlation between Sq and Sdq is not strong. It is the consequence of the large values of the Sal parameter of deterministic surfaces after milling and most of the random-deterministic surfaces after laser texturing. The other random surfaces are characterised by smaller values of the correlation length Sal. Figure 10b shows this dependence after excluding deterministic and strongly random-deterministic textures to highlight the correlation that appears only for truly random surfaces. Results on deterministic milled surfaces (8 samples) and random-deterministic laser-textured surfaces with large contributions from deterministic components (large values of the pit–area ratio and consequently of the Sal parameters—6 samples) were excluded. The number of data points presented in Figure 10b is 50. The coefficient of determination R^2^ between Sq and the Sdq parameters in Figure 10b is 0.74; therefore, the coefficient of linear correlation R is strong, 0.86.

It follows from the analysis of Figure 2, Figure 3, Figure 4, Figure 5, Figure 6, Figure 7, Figure 8 and Figure 9 that the prediction of the Sdr parameter using Formula (3) depends on the surface amplitude. Estimation accuracy was better for smaller slope values (low roughness height and/or high sampling interval). Therefore, the Sdr parameter was accurately predicted for smoother surfaces for the small sampling intervals, but for these SIs, large errors in Sdr parameter estimation occurred for rougher surfaces; these errors were reduced when the SI increased. However, for smother surfaces, the errors in Sdr estimation were high for very large sampling intervals. To study this behaviour, we studied the accuracy of the estimation of the Sdr parameter for various sampling intervals.

**Figure 11:**
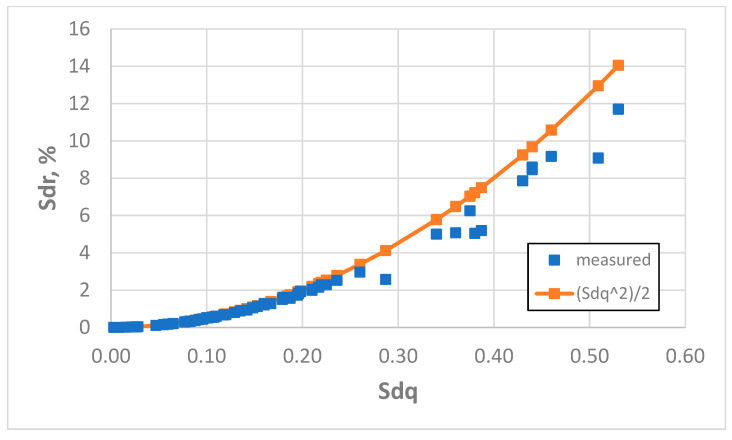
Real and estimated dependence between the Sdq and Sdr parameters, obtained for an initial sampling interval of 3.2 µm.

Figure 11 presents this dependence for an initial sampling interval of 3.2 µm. The coefficient of linear correlation between measured and predicted values of Sdr was 0.992, but this correlation between measured values of the Sdq and Sdr parameters was 0.957. The application of Formula (3) led to an overestimation of the Sdr parameter. The average error in the estimation of the Sdr parameter was 12.2%, and the highest error was 63% on the surface after vapour blasting, characterised by a Sq amplitude parameter of 2.69 µm). Estimation errors higher than 5% were found in 56% of the cases analysed as follows: for three surfaces after milling, all (eight) surfaces after vapour blasting, all (eight) surfaces after vapour blasting followed by lapping, six surfaces after laser texturing, four surfaces after plateau honing and all (eight) surfaces after one-process honing. In 23 cases (36%) the error of Sdr estimation was greater than 10%. Accurate estimations were obtained for smooth surfaces after polishing and lapping (eight surfaces), grinding (eight surfaces), milling (five surfaces), laser texturing (two surfaces) and plateau honing (four surfaces). When the surface amplitude determined by the Sq parameter was less than 0.45 µm, the errors in the estimation of the Sdr parameter using Formula (3) were smaller than 5%. Generally, the application of Formula (3) yields inaccurate results for parameter Sdr estimation for the smallest sampling interval used, except for smooth surfaces.

**Figure 12:**
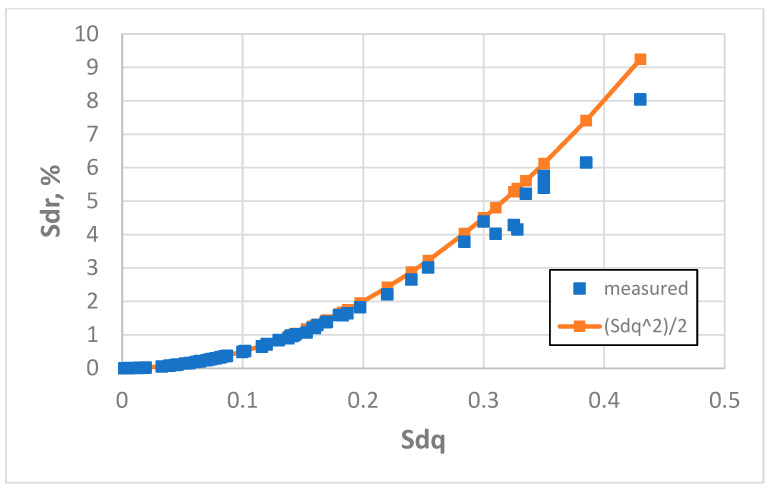
Real and estimated dependence between the Sdq and Sdr parameters, obtained for a sampling interval of 5 µm.

The increase in the sampling interval to 5 µm led to more accurate results of Sdr parameter estimation. The average error was 4.5% and the highest error was 30% (also for the surface after vapour blasting, characterised by a Sq parameter of 2.69 µm). The errors of overestimation of the Sdr parameter were reduced. Relative discrepancies greater than 5% were found for 26% of the analysed surfaces as follows: three after milling, seven after vapour blasting, three after plateau honing, two after one-process honing, and two after vapour blasting followed by lapping. An accurate prediction of the Sdr parameter (errors smaller than 5%) was achieved for all analysed surfaces after polishing or lapping, grinding, and laser texturing. Surface amplitude characterised by a Sq parameter smaller than 1 µm confirmed the results were correct (estimation errors smaller than 5%). An overestimation of the Sdr parameter was found using Formula (3) by more than 10% for three rough surfaces after milling (Sq between 7.8 and 9.1 µm) and three surfaces after vapour blasting (Sq between 2.3 and 2.9 µm). The coefficient of linear correlation between measured and predicted values of the Sdr parameter was 0.997, but between the measured values of Sdq and Sdr it was 0.961. Figure 12 presents the dependence between the Sdq and Sdr parameters for a sampling interval of 5 µm.

**Figure 13:**
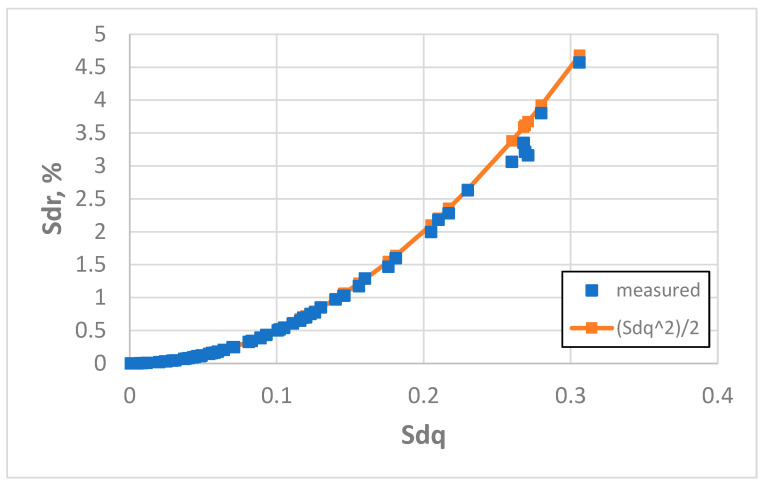
Real and estimated dependence between the Sdq and Sdr parameters, obtained for a sampling interval of 10 µm.

An increase in the sampling interval to 10 µm caused an improvement in the estimation of the Sdr parameter using Formula (3). The mean error was 2.2% and the maximum error was 16.2%. The coefficient of linear correlation between measured and predicted values of the Sdr parameter was 0.9984, but between the measured values of Sdq and Sdr it was 0.958. Only in six cases (9.4%) was the relative error of Sdr estimation greater than 5%. It concerns three surfaces after milling, one surface after vapour blasting, one surface after one-process honing, and one surface after plateau honing; the values of the Sq parameter of these surfaces were greater than 5 µm. In three cases (milled surfaces for the Sq parameter greater than 7.9 µm) the overestimation of the Sdr parameter using Formula 3 was greater than 10% (4.7%). Figure 13 presents the dependence between the Sdq and Sdr parameters for a sampling interval of 10 µm.

**Figure 14:**
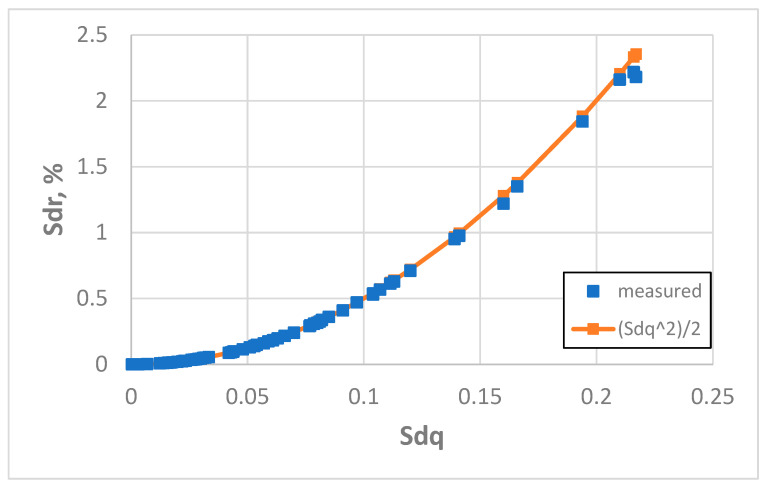
Real and estimated dependence between the Sdq and Sdr parameters, obtained for a sampling interval of 20 µm.

An additional increase in the sampling interval to 20 µm caused an improvement in the estimation of the Sdr parameter using Formula (3). Only in two cases (milled surfaces) were the errors higher than 5% but smaller than 8.1%. The average error was 1.7% and the highest error was 8%. The coefficient of linear correlation between measured and predicted values of the Sdr parameter was 0.9996, but between the measured values of Sdq and Sdr it was 0.949. Figure 14 presents the dependence between the Sdq and Sdr parameters for a sampling interval of 20 µm. A decrease in the coefficient of linear correlation between measured values of the Sdq and Sdr parameters from a sampling interval of 5 µm proved that this dependence was nonlinear in character. An increase in the coefficient of correlation between measured and estimated values of the Sdr parameter confirms Formula (3) better matches the real dependence.

**Figure 15:**
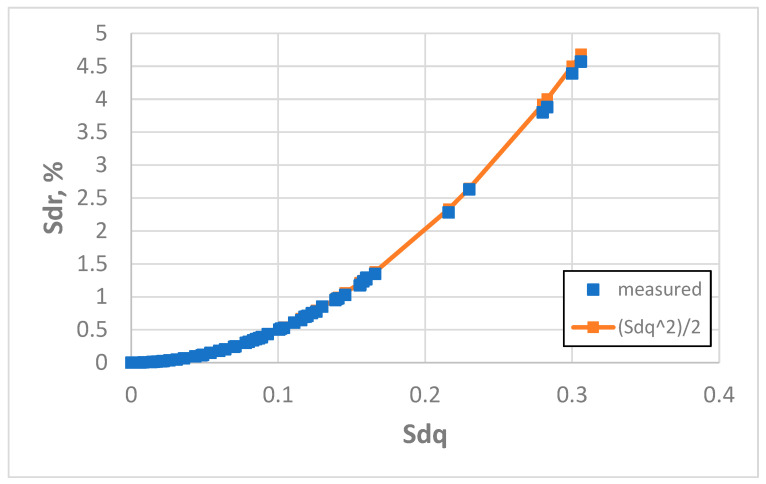
Real and estimated dependence between the Sdq and Sdr parameters, obtained for the recommended sampling intervals.

The application of Formula (3) for the estimation of the Sdr parameter ensured good results for a sampling interval of 20 µm. The further increase in the sampling interval caused higher errors in Sdr estimation for some smooth surfaces. However, sampling intervals of 50 and 100 µm seem to be too large for the used evaluation area (low numbers of measuring points). Therefore, the Sdq–Sdr plots were shown only for sampling intervals of 3.2, 5, 10, and 20 µm. The question arises as to whether an SI of 20 µm is appropriate for all surfaces. The selection of the sampling interval depends on the autocorrelation function. Whitehouse and Archard [26] thought that the sampling interval should correspond to a 0.1 value of autocorrelation length. Other scientists [48,56] recommended a smaller SI. The sampling interval corresponding to a very high correlation between measuring points should be avoided (data are redundant). In our opinion, it should be small enough to adequately estimate the correlation length Sal. So, the SI should be smaller than the initial value of the Sal parameter. A sampling interval of 20 µm is higher than the Sal parameter for some surfaces, especially after polishing, lapping, or grinding. It should be equal to 0.25–0.6 of the Sal parameter, which roughly corresponds to the correlation of measuring ordinates between 0.45 and 0.65 for the exponential shape of the autocorrelation function typical for random surfaces [26]. The recommended values for the SI were typically 5–10 µm for ground surfaces, 20–50 µm for milled surfaces, 10 µm for surfaces after vapour blasting and vapour blasting followed by lapping, 20–50 µm for surfaces after laser texturing, 3.2–5 µm for surfaces after polishing and lapping, 5–10 µm for plateau honed surfaces, and 10 µm for one-process cylinder surfaces. The results suggest that some milled and laser-textured surfaces should be measured with larger evaluation lengths. The increase in the sampling interval caused a decrease in the amplitude Sq parameter and a change (typically an increase) in the correlation length Sal. The average changes in these parameters were 3.5 and 6.5%, respectively. Figure 15 presents the dependence between the Sdq and Sdr parameters for the recommended sampling intervals. The average and maximum errors in the estimation of the Sdr parameter using formula (3) were 1.45 and 3.29%, respectively. The coefficient of linear correlation between measured and predicted values of the Sdr parameter was 0.9999, but between the measured values of the Sdq and Sdr parameters it was 0.938. It attests to the very good estimation of the Sdr parameter using Formula (3) and to the nonlinear dependence between the hybrid parameters Sdq and Sdr.

There are many parameters that describe the texture of areal surfaces. Their number should be reduced. Because the Sdq and Sdr parameters are interrelated, only one hybrid parameter should be selected for surface description. Because the Sdr parameter is more sensitive to measurement errors than Sdq [49], the Sdq parameter is recommended.

## 4. Conclusions

Hybrid parameters are related to the functional properties of machine elements; therefore, their analysis is a task of great significance. The Sdr parameter can be estimated from Sdq. In this paper, the conditions under which the Sdq–Sdr relation is valid were analysed. The effect of the sampling interval on the dependence between the Sdq and Sdr hybrid parameters was studied on the basis of the analysis of a large number of measured surface topographies after various machining treatments. It is difficult to find similar works in the technical literature.

Hybrid parameters strongly depend on the lateral resolution. They decrease with an increase in the sampling interval; therefore, the values of the sampling interval should be presented in the results of surface texture analyses.

The Sdq parameter is strongly correlated with the Sdr parameter. This dependence has a nonlinear character.

The application of the approximating formula caused an overestimation of the Sdr parameter. It can be accurately predicted for small slope values. The slope decreases when the amplitude decreases and the sampling interval increases. Therefore, the estimation of the Sdr parameter based on the values of the Sdq parameter allowed for very accurate results (the errors were smaller than 5%) for smooth surfaces, for which the Sq parameter was smaller than 0.45 µm for a sampling interval no higher than 20 µm. However, for smooth surfaces, errors in Sdr estimation increased for larger sampling intervals. For rougher surfaces, the relative errors in estimating Sdq based on Sdr decreased as the sampling interval increased.

Based on the results of the analysis of all measured surface textures, it was found that relative errors in the estimation of Sdq based on Sdr decreased as the sampling interval increased. For a sampling interval of 5 µm, the estimation inaccuracies were less than 5% when the Sq parameter was smaller than 1 µm. When the Sq parameter was less than 5 µm, the error of estimating Sdr was smaller than 5% when the sampling interval of 10 µm was used. When the sampling interval was 20 µm, relative errors in the prediction of the Sdr parameter were less than 5% for surfaces with a Sq parameter less than 7.5 µm.

Because high sampling intervals do not allow us to obtain accurate values of the spatial parameter Sal, especially of smooth surfaces, the sampling interval, being a fraction of the initial value of the Sal parameter, was recommended. When this interval was used, the errors in Sdr parameter predictions were no higher than 3.3%.

The Sdq–Sdr relation studied is valid for the analysed surfaces measured by a white light interferometer. However, it was found that this dependence ensured accurate results for other surfaces measured by stylus tip profilometers.

Because hybrid parameters are interdependent, one parameter should be selected for the description of the areal surface texture. The Sdq parameter is recommended because of its lower sensitivity to measurement errors than the Sdr parameter.

## Figures and Tables

**Table 1:** Descriptions of analysed surfaces.

Surface	Machining Process
A	grinding
B	milling
C	vapour blasting
D	one-process honing
E	lapping
F	plateau honing
G	vapour blasting followed by lapping
H	laser texturing

**Table 2:** Changes in the hybrid parameters of ground surface A with an increase in the sampling interval.

SI, µm	Sdq	Sdr Measured, %	Sdr Predicted, %	Relative Error
3.2	0.106	0.55	0.5618	−0.02145
5	0.077	0.29	0.29645	−0.02224
10	0.049	0.12	0.12005	−0.00042
20	0.026	0.033	0.0338	−0.02424
50	0.012	0.0069	0.0072	−0.04348
100	0.0057	0.00155	0.001625	−0.04806

**Table 3:** Changes in hybrid parameters of milled surface B with an increase in the sampling interval.

SI, µm	Sdq	Sdr Measured, %	Sdr Predicted, %	Relative Error
3.2	0.36	5.07	6.48	−0.27811
5	0.31	4.02	4.805	−0.19527
10	0.26	3.06	3.38	−0.10458
20	0.21	2.16	2.205	−0.02083
50	0.16	1.266	1.28	−0.01106
100	0.114	0.646	0.6498	−0.00588

**Table 4:** Changes in hybrid parameters of vapour-blasted surface C with an increase in the sampling interval.

SI, µm	Sdq	Sdr Measured, %	Sdr Predicted, %	Relative Error
3.2	0.44	8.59	9.68	−0.12689
5	0.335	5.22	5.61125	−0.07495
10	0.217	2.28	2.35445	−0.03265
20	0.113	0.629	0.63845	−0.01502
50	0.044	0.095	0.0968	−0.01895
100	0.017	0.014	0.01445	−0.03214

**Table 5:** Changes in hybrid parameters of one-process honed surface D with an increase in the sampling interval.

SI, µm	Sdq	Sdr Measured, %	Sdr Predicted, %	Relative Error
3.2	0.2099	2.046	2.202901	−0.07669
5	0.169	1.374	1.42805	−0.03934
10	0.126	0.775	0.7938	−0.02426
20	0.0835	0.335	0.348613	−0.04063
50	0.0418	0.087	0.087362	−0.00416
100	0.015	0.011	0.01125	−0.02273

**Table 6:** Changes in hybrid parameters of lapped surface E with an increase in the sampling interval.

SI, µm	Sdq	Sdr Measured, %	Sdr Predicted, %	Relative Error
3.2	0.0262	0.0342	0.0343	−0.00292
5	0.017	0.0144	0.01445	−0.00347
10	0.0083	0.0034	0.003445	−0.0132
20	0.0033	0.00052	0.000545	−0.04712
50	0.001	0.000047	0.00005	−0.05263
100	0.00046	0.00001	0.0000105	−0.058

**Table 7:** Changes in hybrid parameters of plateau honed surface F with an increase in the sampling interval.

SI, µm	Sdq	Sdr Measured, %	Sdr Predicted, %	Relative Error
3.2	0.095	0.443	0.45125	−0.01862
5	0.064	0.203	0.2048	−0.00887
10	0.038	0.071	0.0722	−0.0169
20	0.02	0.0198	0.02	−0.0101
50	0.0078	0.00297	0.003042	−0.02424
100	0.0028	0.00038	0.000392	−0.03158

**Table 8:** Changes in the hybrid parameters of surface G with an increase in the sampling interval.

SI, µm	Sdq	Sdr Measured, %	Sdr Predicted, %	Relative Error
3.2	0.129	0.798	0.83205	−0.04267
5	0.0999	0.489	0.499001	−0.02045
10	0.071	0.248	0.25205	−0.01633
20	0.043	0.0916	0.09245	−0.00928
50	0.018	0.0161	0.0162	−0.00621
100	0.007	0.00244	0.00245	−0.0041

**Table 9:** Changes in hybrid parameters of textured surface H with an increase in the sampling interval.

SI, µm	Sdq	Sdr Measured, %	Sdr Predicted, %	Relative Error
3.2	0.08	0.31	0.32	−0.03226
5	0.056	0.154	0.1568	−0.01818
10	0.037	0.068	0.06845	−0.00662
20	0.028	0.039	0.0392	−0.00513
50	0.022	0.024	0.0242	−0.00833
100	0.017	0.0144	0.01445	−0.00347

## Data Availability

The original contributions presented in the study are included in the article, and further inquiries can be directed to the corresponding author.
